# The inclusion or exclusion of studies based on critical appraisal results in JBI qualitative systematic reviews: An analysis of practices

**DOI:** 10.1017/rsm.2025.10042

**Published:** 2025-10-23

**Authors:** Romy Menghao Jia, Cindy Stern

**Affiliations:** 1 JBI, School of Public Health, The University of Adelaide, Adelaide, SA, Australia; 2 Health Evidence Synthesis, Recommendations and Impact (HESRI), School of Public Health, The University of Adelaide, Adelaide, SA, Australia

**Keywords:** critical appraisal, evidence synthesis, qualitative systematic reviews

## Abstract

Critical appraisal is a core component of JBI qualitative evidence synthesis, offering insights into the quality of included studies and their potential influence on synthesized findings. However, limited guidance exists on whether, when, and how to exclude studies based on appraisal results. This study examined the methods used in JBI qualitative systematic reviews and the implications for synthesized findings. In this study, a systematic analysis of qualitative reviews published between 2018 and 2022 in *JBI Evidence Synthesis* was conducted. Data on decisions and their justifications were extracted from reviews and protocols. Descriptive and content analysis explored variations in the reported methods. Forty-five reviews were included. Approaches reported varied widely: 24% of reviews included all studies regardless of quality, while others applied exclusion criteria (36%), cutoff scores (11%), or multiple methods (9%). Limited justifications were provided for the approaches. Few reviews cited methodological references to support their decisions. Review authors reported their approach in various sections of the review, with inconsistencies identified in 18% of the sample. In addition, unclear or ambiguous descriptions were also identified in 18% of the included reviews. No clear differences were observed in ConQual scores between reviews that excluded studies and those that did not. Overall, the variability raises concerns about the credibility, transparency, and reproducibility of JBI qualitative systematic reviews. Decisions regarding the inclusion or exclusion of studies based on critical appraisal need to be clearly justified and consistently reported. Further methodological research is needed to support rigorous decision-making and to improve the reliability of synthesized findings.

## Highlights

### What is already known?

- Critical appraisal is a key step in JBI qualitative evidence synthesis, intended to inform confidence in review findings.
- Guidance is limited on whether studies should be excluded based on appraisal results and what criteria should be used for exclusion.

### What is new?

- JBI qualitative systematic reviews use varied approaches to handle critical appraisal results.
- Approaches and justifications are not always clearly reported or aligned across protocols and final reports.
- Few reviews cited references to support their appraisal-related decisions, and ConQual scores did not differ markedly by exclusion strategy.
- There is a need for methodological development to support transparency and reproducibility.

### Potential impact for RSM readers

- Our findings highlight that it is important for reviewers to make informed and deliberate decisions about how to handle critical appraisal results.
- This study promotes greater transparency in qualitative systematic review methods.

## 1 Introduction

Qualitative systematic reviews play a crucial role in evidence-based healthcare by synthesizing high-quality information regarding the perspectives and experiences of patients, caregivers, providers, and other stakeholders by addressing questions related to the usability, meaningfulness, feasibility, and appropriateness of interventions and practices, factors that are essential for implementing holistic and person-centred care. Review findings are used increasingly to support clinical decision-making, health policy development, practice guidelines, and implementation strategies.^1^^,^ ^2^ Different methodologies exist for the synthesis of qualitative findings, such as thematic synthesis, narrative synthesis, meta-ethnography, and meta-aggregation.^3^ The JBI meta-aggregative approach was developed in the early 2000s, led by Professor Alan Pearson.^4^ The approach is grounded in the philosophical principles of pragmatism and aims to generate meaningful recommendations to inform practice.^5^

One fundamental step of JBI qualitative systematic review is critical appraisal.^1^^,^ ^5^^,^ ^6^ Critical appraisal provides an in-depth understanding of the quality of the included studies. In JBI qualitative reviews, critical appraisal is closely linked to the level of confidence that can be placed in synthesized findings.^6^^,^ ^7^ The process is especially critical in JBI meta-aggregative reviews as the findings are intended to inform policy or practice,^5^ where for methodologies focused on generating theoretical insights such as meta-ethnography, the practice remains contentious.^8^^,^ ^9^ Different standards, criteria, checklists, and tools have been developed to assist reviewers in assessing the quality of primary qualitative studies, such as but not limited to the JBI Checklist for Qualitative Research,^1^ the CochrAne qualitative MEthodological LimitatiOns Tool (CAMELOT),^10^ and the Evaluation Tool For Qualitative Studies (ETQS).^11^ The number of available appraisal tools has grown rapidly in recent years, with more than 100 instruments now identified for qualitative research.^12^^,^ ^13^ Critical appraisal using standardized tools is generally superior to *ad hoc* or unclear assessments, as it offers review authors a systematic and transparent approach to evaluating study quality.^10^ The approach to assessing the quality of qualitative studies generally involves determining risks to study rigour or trustworthiness, by looking at the dependability, credibility, and transferability of qualitative studies (Table 1).^14^ Most assessments use a series of criteria that can be scored as being ‘yes’, ‘no’, ‘unclear’, and in some instances ‘not applicable’.

**Table 1:** Criteria for assessing the quality of qualitative research.^14^

Criteria	Definition
Dependability	The research process should be logical, traceable, and clearly documented
Credibility	Addresses whether a finding has been represented correctly
Transferability	Alignment between the situation studied and others to which one might be interested in applying the findings and conclusions of the study

There is ongoing debate regarding which criteria or checklist to use to evaluate qualitative research and whether studies deemed to be of lower methodological quality should be excluded based on appraisal results in qualitative reviews and, if so, what criteria or methods should guide the exclusions, resulting in a lack of agreement in the field.^8^ Historically, one approach has been to exclude studies with lower methodological quality to ensure the findings of a review are based on the ‘best available evidence’.^14^^,^ ^15^According to the current JBI guidance, the decision whether to exclude studies based on critical appraisal results is up to the review team.^5^ Exclusion decisions can be made based on whether a study meets a predetermined proportion of all criteria or whether it satisfies specific criteria. Reviewers can also weight certain criteria differently. Similarly, Cochrane guidance for Cochrane and Campbell reviews states that an assessment of methodological strengths and limitations, whether using a tool or not, can be employed to exclude studies in the sampling stage in the review process.^10^ Review authors may also choose not to exclude studies based on critical appraisal results because qualitative studies with methodological limitations can provide valuable insights, particularly in contexts where evidence is limited, or contribute perspectives from populations that are often underrepresented in the literature.^16^ The ENTREQ statement, a reporting guide for qualitative syntheses, in item 13, states that appraisal results should be presented, indicating which articles, if any, were weighted or excluded based on the assessment, along with the rationale.^17^ While current guidance allows the review team discretion, it is unclear how this is currently undertaken and what implications it has on synthesized results.

The objective of this study was to investigate current practices on the inclusion or exclusion of studies based on critical appraisal results in JBI qualitative systematic reviews. The JBI approach was chosen for this study because appraisal plays a particularly crucial role in JBI meta-aggregation, as the methodology explicitly focuses on informing practice and critical appraisal results directly influence the level of confidence in synthesized findings. This study aims to provide insights that may inform future methodological development within the JBI framework and potentially across the broader evidence synthesis community.

The specific questions addressed are as follows:

- What methods are applied in JBI qualitative systematic reviews to determine the inclusion or exclusion of studies based on critical appraisal results?
- What are the justifications provided for applying these methods?
- Is there a difference in the confidence of synthesized findings between reviews that applied exclusion based on critical appraisal results and those that included all studies regardless of quality?

## 2 Methods

This study included systematic reviews that exclusively included qualitative evidence, reported using the JBI meta-aggregative approach for qualitative synthesis and published in the peer-reviewed journal *JBI Evidence Synthesis* (previously JBI Database of Systematic Reviews and Implementation Reports) between 2018 and 2022. The timeframe was selected to capture the most recent 5 years of publications available at the time of data collection (early 2023). Reviews that included evidence types other than qualitative evidence were excluded to ensure methodological consistency and comparability across the sample.

A full list of qualitative reviews published during the time period was obtained from the *JBI Evidence Synthesis* editorial office (CS is the Deputy Editor-in-Chief of the journal). As part of the editorial process, authors are required to specify the review type (e.g. qualitative), which is then verified by editors, copy editors, and proofreaders during production. While we acknowledge that qualitative systematic reviews using the JBI meta-aggregative approach are also published in other journals, this study focused exclusively on JBI reviews published in *JBI Evidence Synthesis*, as these were required to adhere to JBI standards of conduct and reporting.^18^

### 2.1 Data extraction

Full-text reports of all included reviews were retrieved by RJ. Data extraction was conducted using a standardized data extraction tool (Appendix I, Supplementary Materials). A pilot extraction of five reviews was conducted by both authors independently (RJ and CS) prior to formal data extraction. Upon completion of the pilot, the results and the tool were discussed, and significant modifications were made to the tool. A second round of piloting was then conducted independently by both authors on the same five reviews using the revised tool. Following the second pilot, only minor adjustments were needed. Subsequently, data were extracted from 40 reviews using MS Excel (RJ and CS, 20 reviews each). All data were reviewed by RJ for consistency prior to analysis, with minor modifications made as necessary.

The extracted data included title, year published, country of corresponding author, methods used to address critical appraisal results and their justifications, and the ConQual score. Methods and justifications were extracted verbatim from the review to mitigate any potential for reinterpretation from the authors during data extraction. In addition, a priori protocols for each included review were identified and retrieved where methods pertinent to handling of critical appraisal results were extracted to ensure comprehensive data extraction. References provided in reviews and protocols to support their specified methods and justifications were also extracted.

### 2.2 Data analysis and presentation

Descriptive analysis and basic content analysis were conducted (RJ) to examine the characteristics of the included reviews and methods regarding the inclusion or exclusion of studies based on the methodological quality of studies and justifications.

Specifically, for the characteristics of the included reviews, data were analysed descriptively. Frequency distributions were presented to describe the year and geographical distribution of studies and reporting of methods.

To analyse the methods employed and their justifications, an inductive content analysis was conducted, an approach well suited for exploring phenomena with limited or fragmented prior knowledge.^19^ In this study, while critical appraisal in qualitative evidence synthesis is an established step, there is very limited research specifically examining how review authors make inclusion or exclusion decisions based on appraisal results and how they justify these decisions. Choosing an inductive approach allowed us to explore and categorize patterns in the methods and justifications. The coding process started with reading and re-reading of the extracted data, during which notes were written to describe the methods detailed in each review. The coding framework was developed based on the initial notes. The data were then coded against the framework, with each review being assigned a single code of the method. The frequency of methods was analysed.

During the analysis, it was observed that some review authors described their methods and justification across multiple sections within the review or in both the review and its a priori protocol. In some instances, this resulted in unnecessary repetition, while in others, it led to inconsistencies in the reported information. For example, some protocols indicated that a specific method would be applied, but the corresponding review described a deviation from the methods stipulated a priori. Additionally, where methods for handling of the results of appraisal were described in multiple sections of a review, inconsistencies were identified between the methods reported and appendices, the methods and the results, and the results and the appendices. These inconsistencies were identified in nine reviews, and after discussion between the authors, the following principles were established to handle data:

- If inconsistencies are evident between data extracted from the protocol and review, details extracted from the review will be analysed.
- If inconsistencies are evident between data extracted from the methods and results in the review, details extracted from the results will be analysed.
- If inconsistencies are evident between data extracted from the methods/results and appendices, details extracted from the methods/results will be analysed.

These principles assumed that, compared to the protocol, the review itself offered a more accurate and comprehensive account of the actual conduct regarding appraisal results, acknowledging that the results section of the completed review is considered the most reliable source of information on the applied method.

For reviews where the stated method was inconsistent, only the justifications provided for the analysed method as per the principles defined above were examined. Conversely, when the stated method was consistent throughout the review sections and protocol, justifications provided in all relevant sections (protocol or multiple sections of the review) were included in the analysis.

The analysis of justifications for each method was conducted following the coding of the methods. Reviews were categorized based on their assigned code of method, and justifications were then analysed within each group. Where possible, an inductive content analysis was applied to identify themes in the justifications for each method group. When justification data were limited and insufficient for a method group, verbatim justifications from individual reviews were presented. The frequency of each category was recorded to indicate how many reviews provided justifications.

The association between the confidence of synthesized findings and the method applied was analysed using extracted ConQual scores. In JBI qualitative systematic reviews, synthesized findings are required to be assigned a level of confidence using the ConQual approach,^6^ a process providing essential information for knowledge users when considering using synthesized findings to inform decisions. The JBI ConQual approach is a ranking system for synthesized findings that evaluates the dependability of included studies and credibility of extracted findings included that inform synthesized findings. Credibility is assessed during the data extraction stage by evaluating the congruence between the author’s interpretation and the supporting data. Dependability, on the other hand, is determined using the critical appraisal results from five questions (Q2, Q3, Q4, Q6, and Q7) in the JBI critical appraisal checklist.^1^ This study calculates the number and percentage of synthesized findings classified as high, moderate, low, and very low confidence for each method to examine potential differences in ConQual scores between reviews that applied exclusion criteria and those that did not.

Results are presented in tables and figures with explanations provided to describe the findings.

## 3 Results

### 3.1 Study inclusion

Forty-seven qualitative systematic reviews were published in *JBI Evidence Synthesis* between 2018 and 2022. Two reviews were excluded because their protocols were unavailable despite two attempts to contact the authors. As a result, 45 reviews were included in this study (Appendix II, Supplementary Materials).

### 3.2 Characteristics of included reviews

#### 3.2.1 Year and country of publication

**Figure 1:**
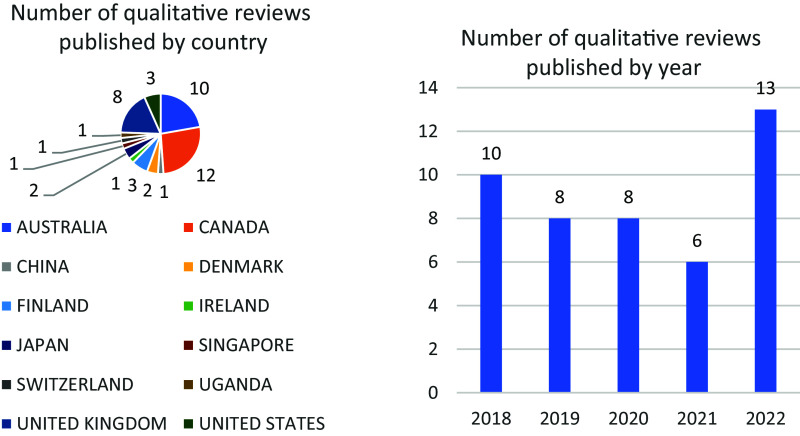
The number of qualitative reviews published by country and year.

Most qualitative reviews were conducted by authors in Canada (12, 27%), Australia (10, 22%), and the UK (8, 18%). The number of published qualitative reviews ranged from 6 in 2021 to 13 in 2022, with other years falling in between (Figure 1).

#### 3.2.2 Reporting of methods regarding the inclusion or exclusion of studies based on methodological quality

**Figure 2:**
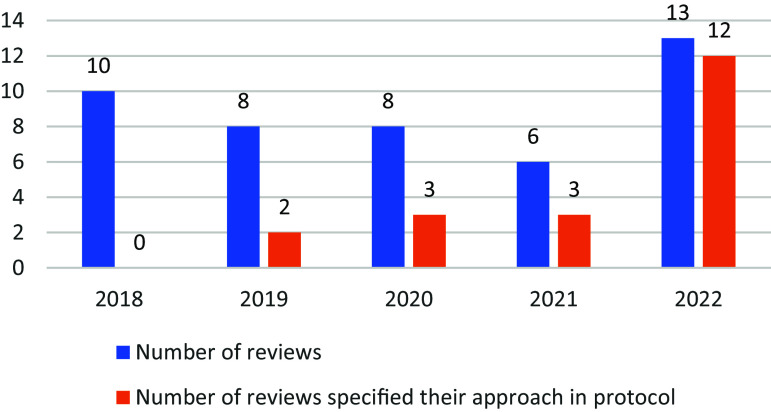
The number of reviews specified a priori methods in the protocol over time.

All 45 included reviews utilized the JBI Checklist for Qualitative Research, in compliance with current JBI guidance for the conduct of JBI qualitative syntheses (Table 2).^1^ Of these, 44 (98%) outlined their method to the inclusion or exclusion of studies based on methodological quality, while one (2%) did not specify their method in either their review or protocol.^20^ Among the 44 (98%) reviews that specified their method, all (98%) provided this information in the review, with 20 (45%) also specifying it in the protocol. Notably, among the 20 that reported the method in their protocol, the majority were published in 2022 (Figure 2). In contrast, none of the reviews published in 2018 reported their methods for handling the results of appraisal in their protocol (Figure 2).

**Table 2:** Methods regarding the inclusion or exclusion of studies based on methodological quality.

Methods	Number of reviews	Percentage (rounded)
Include all studies regardless of methodology quality	11	24%
Certain items in the appraisal checklist deemed essential	16	36%
Cutoff score/percentage utilized	5	11%
Multiple methods utilized	4	9%
Unclear	8	18%
No methods mentioned	1	2%
Total	45	1

**Figure 3:**
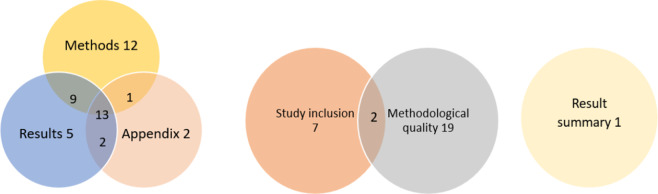
Methods reported in review sections and in results sections.

Of the 44 (98%) reviews that specified their method in the review, 35 (78%) included this information in the methods section, 29 (64%) in the results section, and 18 (40%) in an Supplementary Materials, with 25 (56%) reviews reporting it in more than one section (Figure 3). Among the 29 (64%) reviews that specified their methods in the results section, the majority provided this information in the methodological quality section (21 reviews, 47%), followed by the study inclusion section (9 reviews, 20%). Two reviews (4%) reported their method in both the study inclusion and methodological quality sections, while one review (2%) presented it under the results header instead of subsections. Inconsistent reporting across sections was identified in eight reviews (18%).

### 3.3 Manuscript sections detailing inclusion or exclusion of studies based on methodological quality and justifications

As shown in Table 2, of the 45 included reviews, one (2%) did not report any methods to study inclusion based on methodological quality in either the protocol or review. Eleven reviews (24%) opted to include all studies regardless of the methodological quality. Twenty-five (56%) reviews applied an exclusion-based method, which involved designating certain items on the appraisal checklist as essential (16 reviews, 36%), applying a cutoff score or percentage (5 reviews 11%), or employing a combination of multiple methods (4 reviews, 9%). Eight reviews (24%) mentioned their methods without sufficient clarity (Appendix III, Supplementary Materials).

### 3.4 Include all studies regardless of methodology quality

Of the 11 (24%) reviews that opted to include all studies regardless of methodological quality, eight reviews provided justifications for using this method. One review provided only a reference^21^ alongside its choice.^22^ These justifications broadly aligned with five themes: ensuring a comprehensive understanding of the phenomenon, mitigating the limited number of studies available in the field, considering the impact of methodological quality during the analysis, acknowledging the limitations of the appraisal process, and accounting for the inherent nature of qualitative research (Table 3).

**Table 3:** Justifications for including all studies regardless of methodology quality.

Theme (frequency)	Explanation of the theme	Review reference
Providing a comprehensive understanding of the phenomenon (2)	Including all studies can avoid missing any evidence and provide a richer understanding of the research phenomenon.	^[23, 24]^
Limited studies (2)	The review is focused on an emerging area, and there are a limited number of studies relevant to the phenomena of interest.	^[24, 25]^
Addressed in the analysis (2)	The impact of the methodological quality of the included studies will be considered in the analysis, for example: ConQual scores would provide readers with a level of confidence in the usability of synthesized findings, and the credibility of the synthesis/syntheses can be ensured by excluding unsupported findings before conducting the synthesis.	^[24, 26]^
Limitations in the appraisal process (2)	There are limitations in the appraisal process. The appraisal is based on reviewers’ interpretation of the included data based on limited information. Journal length and editorial requirements may impact the depth of methodological information provided. In addition, using the checklist might compromise the diverse nature of qualitative research.	^[23, 27]^
The nature of qualitative research (3)	Because of the nature of qualitative studies being interpretive and naturalistic, poor-quality studies can also generate potentially valuable insights.	^[24, 28, 29]^

**Table 4:** Exclusion methods.

Exclusion methods	Number of reviews providing justification	Number of reviews	Percentage (rounded)
Certain items in the appraisal checklist deemed essential	6	16	36%
Cutoff score	2	5	11%
Multiple methods utilized	1	4	9%
Total	9	25	56%

There were four references provided across four reviews to support these decisions (Appendix IV, Supplementary Materials). JBI guidance was cited in three reviews.^[21, 30]^ One review cited the work of Leung^31^ and Kisely and Kendall^32^ to highlight limitations in the appraisal process, noting that excluding studies based on appraisal might compromise the diverse nature of qualitative studies. Two reviews referenced Dixon-Woods et al.^33^ to support the argument that even studies of lower quality can provide valuable insights, contributing to a richer understanding of the research phenomenon.

### 3.5 Exclusion methods

Of the 45 included reviews, 25 (56%) opted to apply exclusion-based methods aligned to the results of methodological quality assessment, with the most common being the requirement to fulfil specific items on the appraisal checklist (16 reviews) (Table 4).

#### 3.5.1 Certain items in the appraisal checklist deemed essential

Among the reviews that selected specific items in the appraisal checklist as essential, nearly all (15), with the exception of one,^34^ identified Q8 as a mandatory criterion. Q2 and Q4 were also frequently deemed essential, while Q1 and Q6 were not required by any of the reviews (Table 5). The number of items deemed essential varied, ranging from 1 to 8 (mean 4.1, median 3.5). The combination of items chosen by reviews can be found in Appendix V, Supplementary Materials.

**Table 5:** Frequency of items required in the checklist to determine inclusion/exclusion.

Items in the checklist	Number of reviews	Percentage (rounded)
Q1 Is there congruity between the stated philosophical perspective and the research methodology?	0	0
Q2 Is there congruity between the research methodology and the research question or objectives?	11	24%
Q3 Is there congruity between the research methodology and the methods used to collect data?	8	18%
Q4 Is there congruity between the research methodology and the representation and analysis of data?	11	24%
Q5 Is there congruity between the research methodology and the interpretation of results?	7	16%
Q6 Is there a statement locating the researcher culturally or theoretically?	0	0
Q7 Is the influence of the researcher on the research, and vice versa, addressed?	1	2%
Q8 Are participants, and their voices, adequately represented?	15	33%
Q9 Is the research ethical according to current criteria or, for recent studies, is there evidence of ethical approval by an appropriate body?	4	8%
Q10 Do the conclusions drawn in the research report flow from the analysis, or interpretation, of the data?	7	16%

For the items deemed essential, most reviews (9 out of 16) did not specify what response (i.e. ‘yes’, ‘no’, ‘unclear’, and ‘not applicable’) constituted meeting the criteria. Five reviews explicitly required a ‘yes’ response, while two reviews also accepted ‘unclear’ as sufficient (Appendix VI, Supplementary Materials). One review justified the acceptance of ‘unclear’, arguing that this was necessary to retain a sufficient number of studies with relevant findings of suitable quality to address the review questions and that the assessment of confidence in synthesized findings would inform readers about the level of confidence in findings.^35^

Six reviews provided justifications for their specified method, which were categorized into six themes (Table 6). Three reviews emphasized the importance of ensuring that participants’ voices were adequately captured.^36^^–^ ^38^ One review emphasized that researcher reflexivity is essential to align with accepted standards for reporting qualitative research,^35^ supported by references.^39^^,^ ^40^ Ensuring methodological approaches were appropriate was mentioned in one review.^36^ Another review based its justification on adherence to the decisions made in a previous review protocol.^41^ One review justified the decision in an attempt to optimize confidence in synthesized findings.^42^ One review stated the limited reporting of certain items (Q1 and Q6) in qualitative research as the reason for not selecting them as mandatory.^35^

**Table 6:** Justification for certain items in the appraisal checklist deemed essential.

Theme (frequency)	Explanation of the theme	Review reference
Ensuring that participants’ voices were captured (3)	Certain items such as Q8 were chosen to make sure participants’ voices and experiences were captured.	^[36–38]^
Ensuring methodological approaches were appropriate (1)	No explanation provided.	^[36]^
Addressing researcher reflexivity (1)	To align with reporting guidelines for qualitative research, reflexivity was considered essential.	^[35]^
Adopted method in other reviews (1)	Reviews choose items previously used by another review protocol.^43^	^[41]^
Limited reporting on certain items in studies (1)	The justification for not selecting certain items, such as Q1 and Q6, was that these items are often not reported in studies.	^[35]^
Optimized confidence in synthesized findings (1)	Items related to dependability and credibility score in ConQual were chosen to optimize confidence in synthesized findings.	^[42]^

**Table 7:** Cutoff score details per review.

Minimum				
number of				
items	Required answer			Review
required	(yes, no, unclear)	Scoring system	Justification (verbatim)	reference
8	Yes	Did not specify	‘to ensure that only rigorous research informs the synthesized findings of this review’	^[44]^
8	Did not specify	Each item was assigned a value of 1	Not provided	^[45]^
7	Yes	All criteria were given equal value	‘This cutoff score was considered to ensure high quality studies were included in the review’	^[46]^
5	Only ‘yes’ will be given a score of 1	Yes, score 1; unclear, score 0. Not applicable, this point was subtracted from the total score	Not provided	^[47]^
5	Yes	Did not specify	Not provided	^[48]^

#### 3.5.2 Cutoff score

Five reviews applied a cutoff score to determine inclusion/exclusion(Table 7). Two reviews required eight items, one review required seven items, and another required five items to be satisfied for inclusion. One review, instead of specifying the number of required items, set a minimum overall score of 5 based on its scoring system. In terms of the required responses to these questions, four reviews specified that only ‘yes’ responses were acceptable, while the other review did not specify it. Three reviews explicitly stated that all items were weighted equally, while two did not provide this information. One review also specified the score for ‘unclear’ and ‘not applicable’ responses, being 0 for ‘unclear’ and subtracting ‘not applicable’ responses from the total score. Two reviews provided justifications for applying a cutoff score, both emphasizing the importance of ensuring that only high-quality studies inform synthesized findings.

#### 3.5.3 Multiple methods utilized

Four reviews employed multiple methods (Table 8). Two reviews utilized a cutoff score and team discussions. Both required studies to meet ‘yes’ responses for eight items, and for studies that did not meet this criterion, it was stated that reviewers will meet and discuss their inclusion. One of the two reviews also reported that they decided to include a study that did not reach the cutoff because ‘it provided good findings’.^49^

**Table 8:** Multiple methods reported per review.

Requirement	Required answer (yes, no, unclear)	Justification (verbatim)	Review reference
80% and discussion	Yes	Did not provide	^[49]^
80% and discussion	Yes	Did not provide	^[50]^
70% + checklist items 2, 3, 4, 5, and 8	Did not specify	Did not provide	^[51]^
50% + checklist item 8	Yes	Based on the evaluation of the first five included studies by three reviewers	^[52]^

**Table 9:** The number and percentage of high-, moderate-, low-, and very low-confidence synthesized findings for each method identified.

	Total number of synthesized findings across reviews (*n*)	Total number of high-confidence synthesized findings across reviews (*n*, %)	Total number of moderate-confidence synthesized findings across reviews (*n*, %)	Total number of low-confidence synthesized findings across reviews (*n*, %)	Total number of very low-confidence synthesized findings across reviews (*n*, %)
Include all	52	5 (10%)	27 (52%)	16 (31%)	4 (8%)
regardless of
methodology
quality
Cutoff score	20	0	8 (40%)	8 (40%)	4 (20%)
Certain items	67	10 (15%)	33 (49%)	24 (36%)	0
deemed
essential
Multiple	19	0	11 (58%)	8 (42%)	0
methods
utilized

*Note:* Percentages may not total 100% due to rounding.

Another two reviews required a certain percentage of items to be met while also setting additional eliminatory criteria (Table 8). Only one review provided a justification for using multiple methods, explaining that this decision was informed by an evaluation of the first five included studies conducted by three reviewers.

### 3.6 ConQual score by methods

There were no apparent differences in ConQual scores between reviews that applied exclusion criteria and reviews that included all studies regardless of methodology quality. There are 158 synthesized findings in total across the 36 reviews with a clearly categorized method, excluding those that did not report their method (one review) or provided unclear descriptions (eight reviews) (Table 2). As shown in Table 9, reviews using the exclusion methods based on certain items in the checklist had the highest proportion of high-confidence synthesized findings (15%). For moderate-confidence synthesized findings, the exclusion method using multiple methods had the highest proportion (58%). Reviews that employed multiple methods showed the highest percentage of low-confidence synthesized findings (42%), while those using the cutoff score method reported the highest proportion of very low-confidence findings (20%).

## 4 Discussion

The findings of this study demonstrate variations in methods applied in JBI qualitative systematic reviews regarding the inclusion or exclusion of studies based on methodological quality. About one-third of the reviews chose to include studies regardless of methodological quality. This approach was justified by providing a comprehensive understanding of the phenomenon or the limited number of available studies.^23^^–^ ^25^ Some reviews suggested that the quality concerns of the included studies could be addressed in the analysis process, either by the elimination of unsupported findings during data extraction or via ConQual, which informs the readers of the confidence they can place on synthesized findings.^24^^,^ ^53^ It is unsurprising that authors make connections between critical appraisal and ConQual, as the answers for five questions in the critical appraisal tool inform the ConQual assessment.

It is worth noting that some authors also outlined methodological concerns for excluding studies based on the critical appraisal results due to limitations in the appraisal process. They suggested that appraisal is based on reviewers’ interpretation of the included data, and in-depth methodological details are often limited due to journal length and editorial requirements.^23^ There were also reviewers who argued that poor-quality qualitative studies can provide valuable findings^24^^,^ ^28^ and expressed concern that using the checklist might compromise the diverse nature of qualitative research, suggesting further development of the appraisal framework.^27^ This is an area of ongoing debate historically as appraisal is a convention for quantitative studies, which prioritize standardization and objectivity, whereas qualitative research is context-dependent, interpretative, and grounded in naturalistic inquiry,^31^^,^ ^54^ making it less suited for rigid evaluation criteria. These concerns raise valid questions about the suitability of using appraisals as a basis for excluding studies in qualitative reviews.

In contrast, the majority of reviews applied exclusion parameters, with the most common method being the selection of specific checklist items that were deemed essential. Although the items selected and their combinations varied, most of them reflected an emphasis on ensuring participants’ voices were captured. One review justified this decision by referencing a previous review,^41^ highlighting the limited methodological research and guidance available in this area. Interestingly, instead of leaving the methodological concerns to be addressed in ConQual, there was a review that chose criteria required by ConQual as mandatory to optimize confidence in synthesized findings.^42^ However, it remains unclear whether selecting criteria aligned with ConQual necessarily results in high-confidence findings as anticipated, which warrants further investigation in future research. In addition to Q8, questions that assess the congruity between the research methodology and the research question (Q2), as well as the representation and analysis of data (Q4), were also commonly applied.

Methods using cutoff scores primarily aimed to include only high-quality studies, and a small subset of reviews adopted multiple methods. An interesting justification involved reviewers evaluating the first few included studies to inform their methods;^52^ however, limited details on the implementation of these methods were reported. One review appeared to comment on their method based on appraisal results, suggesting that inclusion decisions may have been made after evaluating appraisal results rather than following a predefined strategy.^55^

The findings indicate no clear differences in ConQual scores between reviews that applied exclusion criteria based on methodological quality and those that included all studies regardless of quality. Our initial hypothesis was that excluding lower-quality studies would lead to higher ConQual scores. However, confirming this would require investigating critical appraisal results and reintroducing the excluded studies in reviews, reassessing the synthesized findings, and comparing the resulting ConQual scores, which is beyond the scope of this study. Future research is encouraged to explore this issue further. For example, a Study Within a Review (SWAR) could be conducted to examine how different approaches to handling appraisal results influence the ConQual score within the same review.^56^ The findings of this study also found a lack of clarity in the reporting of methods used in JBI qualitative systematic reviews related to the inclusion or exclusion of studies based on critical appraisal results. Although most reviews (44 of 45) outlined a method, there were eight cases (Appendix III, Supplementary Materials) where the reporting was unclear, and the specific method applied could not be determined. Among the 36 where the method could be identified, only 17 provided justifications, many of which were brief and minimal. This aligns with observations in the existing literature, which have similarly highlighted the limited reporting of methods in qualitative reviews.^33^ Inconsistencies were found between protocols and different sections of individual reviews. Only a few reviews provided justifications for their chosen methods, and the justifications often lacked sufficient detail. Even fewer reviews cited references to support their decisions, highlighting a lack of methodological research and guidance in this area.

Overall, there are substantial inconsistencies in the approaches taken and the rationales provided. At this stage, it may not be possible to make a definitive recommendation about whether studies should always be included or excluded based on appraisal results, under what conditions studies should be excluded, and suggesting criteria or decision rules for applying exclusion thresholds or essential appraisal items. And it remains uncertain whether consistency in the methodological choices is needed—or even reasonable to expect—given the unique nature of qualitative evidence, the ongoing debate around appraisal in qualitative research, and the unclear implications for confidence in synthesized findings. We recommend targeted methodological research to examine the implications of different appraisal-related decisions and theoretical development on the alignment between these decisions and the philosophical underpinnings of the methodology, which is critical for determining whether greater methodological consistency is desirable or necessary. In addition, clear, consistent, and transparent reporting is essential. Poor reporting potentially impacts the transparency and reproducibility of the review and makes it difficult to assess how appraisal decisions are being applied in practice. This study has several limitations that should be acknowledged. First, the scope of the review was restricted to publications up to 2022. Additional reviews have since been published. However, there were no substantive changes in JBI methodological guidance on the inclusion or exclusion of studies based on critical appraisal outcomes until late 2024.^5^ Consequently, the evidence synthesized from this period reflects current methodological practice and remains applicable, ensuring that the findings of this study are both timely and relevant. According to the 2024 updated guidance,^5^ reviewers are expected to provide a detailed rationale regarding which criteria were used and why particular criteria were selected as key markers of study quality. Future research is encouraged to examine how reviews published after this update address these requirements. Second, the analysis was limited to JBI qualitative systematic reviews, meaning the findings may not be generalizable to reviews conducted using other methodologies or frameworks. Future work is encouraged to explore practices across different qualitative synthesis methodologies. Third, the limited and often unclear reporting of methods made it challenging to determine with certainty the methods applied in some reviews. Finally, the principles used to address inconsistencies between protocols and different sections of reviews were based on the authors’ assumption that the review itself provides the most accurate account of how critical appraisal results were addressed, with the results section being considered the most reliable source. This assumption, while practical, may not fully capture the complexities of the decision-making processes documented across various sections or in the protocol.

## 5 Conclusion

This study demonstrates that JBI qualitative systematic reviews employ a variety of methods to determine the inclusion or exclusion of studies based on critical appraisal results. These methods range from including all studies irrespective of their methodological quality to applying exclusion criteria based on specific appraisal items, cutoff scores, or a combination of methods. The findings highlight the challenges in addressing critical appraisal results within qualitative evidence synthesis and the limited and inconsistent reporting of these methods. Additionally, no clear differences in ConQual scores were found between reviews that applied exclusion criteria and those that included all studies regardless of methodological quality. Future research is encouraged to investigate this issue further to better understand its implications. Addressing these gaps in future research in this area is essential for ensuring more robust and transparent qualitative systematic reviews.

## Supporting information

Jia and Stern supplementary material Jia and Stern supplementary material

## Data Availability

The data used for analysis are publicly available at https://doi.org/10.25909/30125557.v2.

## Supplementary material

To view supplementary material for this article, please visit http://doi.org/10.1017/rsm.2025.10042.
