# Supplementary material for: The inclusion or exclusion of studies based on critical appraisal results in JBI qualitative systematic reviews: An analysis of practices
Source: Res Synth Methods. 2025 Oct 23;17(2):277–92. doi: 10.1017/rsm.2025.10042 (PMC12873616; doi:10.1017/rsm.2025.10042)
Supplement: Jia and Stern supplementary material [file S1759287925100422sup001.zip › S1759287925100422sup001/Appendix I Data extraction tool.docx]

**Appendix I: Data extraction tool**

|  | **Basic characteristic** | | |
| --- | --- | --- | --- |
|  | Manuscript Number |  |  |
|  | Title |  |  |
|  | Year Published |  |  |
|  | Country | Country of correspondence author |  |
|  | Review question(s) |  |  |
|  | Critical appraisal tool used |  |  |
|  | ConQual- Number of synthesized findings | Number of synthesized findings |  |
|  | ConQual- Number of high, low, very low synthesized finding | Number of high, low, very low synthesized finding |  |
|  | **Protocol** | | |
| 1 | Was the method regarding the inclusion or exclusion of studies based on methodological quality specified in the protocol? | - Yes - No method specified (instead of no) |  |
| 1a | If yes, please describe the method (verbatim) | - Verbatim extraction - NA for those answered No method specified for question 1 |  |
| 1b | If yes, did the protocol provide a justification for the method specified? What is the justification? | - Yes- Verbatim extraction of the justification. If there is a reference cited, extract the reference. - No justification provided (instead of no)--- for those ones have specified method but did not provide any justification - NA for those answered No method specified for question 1 | The justifications are why they choose the specified method instead of the method itself. |
| **Review** | | | |
| 2 | Did the review specify the method in the methods section regarding the inclusion or exclusion of studies based on methodological quality? | - Yes - No method specified in methods |  |
| 2a | If yes, please extract the method (verbatim) | - Verbatim extraction - NA for those answered No method specified in methods for question 2 |  |
| 2b | If yes, did the review provide a justification for specified method? What is the justification? | - Yes- Verbatim extraction of the justification. If there is a reference cited, extract the reference - No justification provided (instead of no)--- for those ones have specified method but did not provide any justification - NA for those answered No method specified in methods for question 2 |  |
| 3 | Did the review specify the method in the result section regarding the inclusion or exclusion of studies based on methodological quality? | - Yes (specify the section title, for example, methodological quality of included studies, characteristic of included studies) - No method specified in results | Only answer yes when specifics about the method were provided |
| 3a | If yes, please extract the method (verbatim) | - Verbatim extraction - NA for those answered No method specified in results for question 3 |  |
| 3b | If yes, did the review provide a justification for specified method? What is the justification? | - Yes- Verbatim extraction of the justification. If there is a reference cited, extract the reference - No justification provided (instead of no)--- for those ones have specified method but did not provide any justification - NA for those answered No method specified in results for question 3 |  |
| 4 | Did the review specify the reasons for exclusion of individual studies during appraisal in the appendix? If yes, please extract the data verbatim | - Yes-- Verbatim extraction - No reasons specified in appendix - No study excluded |  |
| 5 | Extractor’s comment | - Highlight here when the review address inclusion criteria |  |
