# Supplementary material for: The inclusion or exclusion of studies based on critical appraisal results in JBI qualitative systematic reviews: An analysis of practices
Source: Res Synth Methods. 2025 Oct 23;17(2):277–92. doi: 10.1017/rsm.2025.10042 (PMC12873616; doi:10.1017/rsm.2025.10042)
Supplement: Jia and Stern supplementary material [file S1759287925100422sup001.zip › S1759287925100422sup001/Appendix II Reviews included in this study.docx]

| **Author Year** | **Title** |
| --- | --- |
| Forough et al., 2018 | Nurse experiences of medication administration to people with swallowing difficulties living in aged care facilities: a systematic review of qualitative evidence |
| Small et al., 2018 | Experiences and cessation needs of Indigenous women who smoke during pregnancy: a systematic review of qualitative evidence |
| Jessen-Winge et al., 2018 | The influence of occupation on wellbeing, as experienced by the elderly: A systematic review |
| Baldwin et al., 2018 | Mental health and wellbeing during the transition to fatherhood: a systematic review of first time fathers' experiences |
| Carrier et al., 2018 | Men's perceptions of the impact of the physical consequences of a radical prostatectomy on their quality of life: a qualitative systematic review |
| Parsons et al., 2018 | Older nurses' experiences of providing direct care in hospital nursing units: a qualitative systematic review |
| Lloyd et al., 2018 | Experiences of stroke survivors, their families and unpaid carers in goal setting within stroke rehabilitation: a systematic review of qualitative evidence |
| Petersen et al., 2018 | Scandinavian women's experiences with abortions on request: a systematic review |
| Tay et al., 2018 | Experiences of adult cancer patients receiving counseling from nurses: a qualitative systematic review |
| Carey et al., 2018 | Experiences of undergraduate nursing students in peer assisted learning in clinical practice: a qualitative systematic review |
| McCann et al., 2019 | Sexuality and intimacy among people with serious mental illness: a systematic review of qualitative research |
| Hunter et al., 2019 | Experiences of people with Parkinson's disease and their views on physical activity interventions: a qualitative systematic review |
| Holopainen & Hakulinen, 2019 | New parents' experiences of postpartum depression: a systematic review of qualitative evidence |
| Siltanen et al., 2019 | Family members’ experiences and expectations of self-management counseling while caring for a person with chronic obstructive pulmonary disease: a systematic review of qualitative evidence |
| Salmond et al., 2019 | Experiences of compassion fatigue in direct care nurses: a qualitative systematic review |
| McCloskey et al., 2019 | Patient, family and nurse experiences with patient presence during handovers in acute care hospital settings: a systematic review of qualitative evidence |
| Norberg et al., 2019 | Experiences of HIV-infected adults and healthcare providers with healthcare delivery practices that influence engagement in US primary healthcare settings: a qualitative systematic review |
| Fawcett et al., 2019 | Experiences of parents and carers in managing asthma in children: a qualitative systematic review |
| Picton et al., 2020 | Experiences of outdoor nature-based therapeutic recreation programs for persons with a mental illness: a qualitative systematic review |
| Sbragia & Vottero, 2020 | Experiences of transgender men in seeking gynecological and reproductive health care: a qualitative systematic review |
| Wang et al., 2020 | Barriers and facilitators to physical activity among ethnic Chinese children: a qualitative systematic review |
| Keeping-Burke et al., 2020 | Nursing students’ experiences with clinical placement in residential aged care facilities: a systematic review of qualitative evidence |
| Didier et al., 2020 | Patients’ perspectives on interprofessional collaboration between health care professionals during hospitalization: a qualitative systematic review |
| Barnes et al., 2020 | Health professionals' experiences of grief associated with the death of pediatric patients: a systematic review |
| Macdonald et al., 2020 | Experiences and perceptions of spousal/partner caregivers providing care for community-dwelling adults with dementia: a qualitative systematic review |
| Pike et al., 2020 | The experiences of adults living with an implantable cardioverter defibrillator for cardiovascular disease: a systematic review of qualitative evidence |
| Ramasamy et al., 2021 | Experiences of adults with intellectual disabilities who identify as lesbian, gay, bisexual or transgender within mainstream community: a systematic review of qualitative studies |
| Cusack & Smith, 2021 | Experiences of women discharged early post-vaginal birth: a qualitative systematic review |
| Nixon et al., 2021 | Barriers and facilitators to type 2 diabetes management in the Caribbean region: a qualitative systematic review |
| Parsons et al., 2021 | Experiences of older adults accessing specialized health care services in rural and remote areas: a qualitative systematic review |
| Hanna et al., 2021 | Experiences of learning, development, and preparedness for clinical practice among undergraduate paramedicine students, graduate/intern paramedics, and their preceptors: a qualitative systematic review |
| Kynoch et al., 2021 | Experiences and needs of families with a relative admitted to an adult intensive care unit: a systematic review of qualitative studies |
| Tuomikoski et al., 2022 | Experiences of people with progressive memory disorders participating in non-pharmacological interventions: a qualitative systematic review |
| Bayo et al., 2022 | Mothers’ perceptions of the practice of kangaroo mother care for preterm neonates in sub-Saharan Africa: a systematic review of qualitative evidence |
| McCloskey et al., 2022 | Experiences of faculty and staff nurses working with nursing students during clinical placement in residential aged care facilities: a systematic review of qualitative evidence |
| Parsons et al., 2022 | Nurse leaders’ experiences of upwards violence in the workplace: a qualitative systematic review |
| Cramm et al., 2022 | Experience of children growing up with a parent who has military-related post-traumatic stress disorder: a qualitative systematic review |
| Hassanein et al., 2022 | Experiences and views of parents on second-hand smoke exposure prevention in Middle Eastern countries: a qualitative systematic review |
| Davenport et al., 2022 | Fathers’ experience of depression during the perinatal period: a qualitative systematic review |
| Kassam et al., 2022 | Experiences of nurses caring for involuntary migrant maternal women: a qualitative systematic review |
| Tanywe et al., 2022 | Perceptions and practices of community members relating to trachoma in Africa: a qualitative systematic review |
| Min et al., 2022 | Experiences of living with Juvenile Idiopathic Arthritis: a qualitative systematic review |
| Maehara et al., 2022 | Experiences of transition to motherhood among pregnant women following assisted reproductive technology: a qualitative systematic review |
| Koto et al., 2022 | Experiences of patients with lysosomal storage disorders who are receiving enzyme-replacement therapy and the experiences of their family members: a qualitative systematic review |
| Small et al., 2022 | Barriers to and facilitators of labor market engagement for individuals with chronic physical illnesses in their experiences with work disability policy: a qualitative systematic review |
