# Supplementary material for: The inclusion or exclusion of studies based on critical appraisal results in JBI qualitative systematic reviews: An analysis of practices
Source: Res Synth Methods. 2025 Oct 23;17(2):277–92. doi: 10.1017/rsm.2025.10042 (PMC12873616; doi:10.1017/rsm.2025.10042)
Supplement: Jia and Stern supplementary material [file S1759287925100422sup001.zip › S1759287925100422sup001/Appendix III Reasons for unclear method.docx]

| **Review Source** | **Reason** |
| --- | --- |
| - Petersen M, Jessen-Winge C, Mobjerg A. Scandinavian women's experiences with abortions on request: a systematic review. 2018;16(7):1537-1563. doi:10.11124/JBISRIR-2017-003344. - Carey M, Kent B, Latour J. Experiences of undergraduate nursing students in peer assisted learning in clinical practice: a qualitative systematic review. 2018;16(5):1190-1219. doi:10.11124/JBISRIR-2016-003295. - Parsons K, Gaudine A, Swab M. Older nurses' experiences of providing direct care in hospital nursing units: a qualitative systematic review. 2018;16(3):669-700. doi:10.11124/JBISRIR-2017-003372. | Methods provided in appendix re the reason for exclusion after appraisal for individual studies. Unable to determine the method applied to all studies in the review. |
| - Baldwin S, Malone M, Sandall J, Bick D. Mental health and wellbeing during the transition to fatherhood: a systematic review of first time fathers' experiences. 2018;16(11):2118-2191. doi:10.11124/JBISRIR-2017-003773 - Davenport C, Lambie J, Owen C, Swami V. Fathers’ experiences of depression during the perinatal period: A qualitative systematic review. JBI evidence synthesis. 2022;20(9):2244-2302. - Jessen-Winge C, Petersen M, Morville A-L. The influence of occupation on wellbeing, as experienced by the elderly: a systematic review. 2018;16(5):1174-1189. doi:10.11124/JBISRIR-2016-003123. | Commented on criteria related to study quality in the results but did not specify the criteria used. |
| McCann E, Donohue G, de Jager J, Nugter A, Stewart J, Eustace-Cook J. Sexuality and intimacy among people with serious mental illness: a qualitative systematic review. 2019;17(1):74-125. doi:10.11124/JBISRIR-2017-003824. | Mentioned all studies met key criteria without specification of what the key criteria were. |
| Kynoch K, Ramis M-A, McArdle A. Experiences and needs of families with a relative admitted to an adult intensive care unit: a systematic review of qualitative studies. JBI evid synth. 2021;19(7):1499-1554. doi:10.11124/JBIES-20-00136. | Studies excluded after appraisal due to limited data not related to appraisal. |
