# Supplementary material for: The inclusion or exclusion of studies based on critical appraisal results in JBI qualitative systematic reviews: An analysis of practices
Source: Res Synth Methods. 2025 Oct 23;17(2):277–92. doi: 10.1017/rsm.2025.10042 (PMC12873616; doi:10.1017/rsm.2025.10042)
Supplement: Jia and Stern supplementary material [file S1759287925100422sup001.zip › S1759287925100422sup001/Appendix IV References provided in reviews to support the specified method and justification.docx]

| **References provided in reviews to support the specified method and justification** | **Number of reviews** | **Review Source** |
| --- | --- | --- |
| Lockwood C, Porrit K, Munn Z, et al. Chapter 2: systematic reviews of qualitative evidence. In: Aromataris E, Munn Z, eds. Joanna Briggs Institute Reviewer’s Manual. Joanna Briggs Institute; 2017.  Lockwood C, Porrit K, Munn Z, et al. Chapter 2: systematic reviews of qualitative evidence. In: Aromataris E, Munn Z, eds. JBI Manual for Evidence Synthesis JBI; 2020. | 3 | Tuomikoski et al., 2022;  Nixon et al., 2021  Wang et al., 2020 |
| Dixon-Woods M, Booth A, Sutton AJ. Synthesizing qualitative research: a review of published reports. *Qualitative Research*. 2007;7(3):375-422. | 2 | Nixon et al., 2021  Wang et al., 2020 |
| Leung L. Validity, reliability, and generalizability in qualitative research. *Journal of Family Medicine and Primary Care*. 2015;4(3) | 1 | Ramasamy et al., 2021 |
| Kisely S, Kendall E. Critically appraising qualitative research: A guide for clinicians more familiar with quantitative techniques. *Australasian Psychiatry*. 2011;19(4):364-367. | 1 | Ramasamy et al., 2021 |
